# Fast insights into chitosan-cleaving enzymes by simultaneous analysis of polymers and oligomers through size exclusion chromatography

**DOI:** 10.1038/s41598-024-54002-2

**Published:** 2024-02-10

**Authors:** Margareta J. Hellmann, Bruno M. Moerschbacher, Stefan Cord-Landwehr

**Affiliations:** Institute for Biology and Biotechnology of Plants, University of Münster, 48143 Münster, Germany

**Keywords:** Hydrolases, Carbohydrates, Mass spectrometry, Liquid chromatography

## Abstract

The thorough characterization of chitosan-cleaving enzymes is crucial to unveil structure–function relationships of this promising class of biomolecules for both, enzymatic fingerprinting analyses and to use the enzymes as biotechnological tools to produce tailor-made chitosans for diverse applications. Analyzing polymeric substrates as well as oligomeric products has been established as an effective way to understand the actions of enzymes, but it currently requires separate, rather laborious methods to obtain the full picture. Here, we present ultra high performance size exclusion chromatography coupled to refractive index and mass spectrometry detection (UHPSEC-RI-MS) as a straightforward method for the semi-quantitative analysis of chitosan oligomers of up to ten monomers in length. Additionally, the method allows to determine the average molecular weight of the remaining polymers and its distribution. By sampling live from an ongoing enzymatic reaction, UHPSEC-RI-MS offers the unique opportunity to analyze polymers and oligomers simultaneously—i.e., to monitor the molecular weight reduction of the polymeric substrate over the course of the digestion, while at the same time analyzing the emerging oligomeric products in a semi-quantitative manner. In this way, a single simple analysis yields detailed insights into an enzyme’s action on a given substrate.

## Introduction

Chitosans are a family of polysaccharides that attract increasing interest for diverse applications. They are industrially produced through partial chemical deacetylation of chitin, a linear polymer consisting of β-1,4-linked *N*-acetyl-d-glucosamine units (GlcNAc, A-units) which can be found in the exoskeletons of crustaceans and insects, and in the cell walls of fungi^1^. The partial removal of* N*-acetyl groups converts the water insoluble chitin into soluble chitosans^2^ composed of β-1,4-linked GlcNAc and d-glucosamine units (GlcN, D-units). These linear copolymers have diverse structures which can be characterized by three key factors^3^: The degree of polymerization (DP) describes the number of monosaccharide moieties to form a chitosan molecule; the proportion of GlcNAc units and their distribution along the sugar’s chain are termed fraction of acetylation (FA) and pattern of acetylation (PA), respectively. Fields for applications of these glycans range from agriculture^4,5^ over drug or gene delivery in nanoparticles^6^ to water purification^7,8^, wound healing^9–11^, food preservation^12^, and cosmetics^13^. This high diversity in applications and activities is linked to the structural diversity of chitosans, which in turn is based on the natural variety of chitosan-synthesizing, -cleaving and -modifying enzymes that influence DP, FA, and PA.

A wide spectrum of chitosan hydrolyzing enzymes has been described, each preferring substrates and releasing products of certain DP, FA, and PA. Crucial factors for these differences are the shape of the catalytic site, and the preferences for A- and D-units at the enzyme’s individual subsites, each binding one sugar unit. A widely accepted terminology exists for glycan-cleaving enzymes: Subsites which bind the substrate’s subunits towards its non-reducing end from the hydrolyzed bond are referred to as subsites − 1, − 2, and so on; subsites binding towards the reducing end are subsites + 1, + 2, and so forth^14^. Chitinases^15–17^ prefer A-units at most subsites—their activity increases with increasing FA. In contrast, the activity of chitosanases^18^ decreases with increasing FA, because they favor D-units over A-units at most subsites.

On the one hand, the characterization of chitosan hydrolyzing enzymes is crucial to unravel structure–function relationships of chitosans in vivo, thereby providing insights into the enzymes’ biological role. On the other hand, well-characterized enzymes can be exploited as biotechnological tools to produce tailor-made chitosans that exhibit optimal performance in specific applications. However, the characterization of a chitinase^15–17^, chitosanase^18^, or chitinosanase^19^ is a laborious process that involves a thorough analysis of the chitosan substrates and the enzymatic products, at least concerning DP and FA. For polymeric substrates, the gold standards are nuclear magnetic resonance (NMR) spectroscopy to determine the average FA^20^, and size exclusion chromatography coupled to multiangle light scattering and refractive index detection (SEC-MALS-RI)^3,21^ to analyze the molecular weight (MW). The latter allows the calculation of the DP if the FA is known. Apart from the MW distribution plot, the method provides values for the weight average MW (*M*_w_), number average MW (*M*_n_), and dispersity (*Đ*_M_)—for details about the calculation and significance of these three quantities, as well as the distinction between *M*_w_ and *M*_n_, see the supplementary information.

Both NMR spectroscopy and SEC-MALS-RI need individual sample preparation and the former requires substantial amounts of chitosan polymer in the milligram range. The obtained results are evaluated using integration, whereby the definition of integral start and end can be challenging for samples with low signal strength and/or strong noise. Recently, enzymatic fingerprinting using detection by mass spectrometry (MS) has been proposed as a new method to determine the average FA of polymers that uses only microgram amounts of sample and produces robust, objective results^22^. In contrast, no optimized alternatives to SEC-MALS-RI are available for MW analysis.

Of even greater importance for enzyme characterization is the analysis of small enzyme products, the released non-, partially, or fully acetylated chitooligosaccharides (COS). Whereas these can be easily annotated in terms of DP and FA based on their m/z values obtained by MS, different COS have different signal response factors, making quantification by MS challenging. Currently, quantitative MS sequencing^23^ is the most sophisticated method, allowing even absolute quantification of oligomers of a certain DP, FA, and PA with high accuracy. However, it requires derivatization of the samples in the form of complete chemical *N*-acetylation using isotope-labeled acetic anhydride, followed by reducing end ^18^O-labeling of MS^2^ samples and addition of defined amounts of double isotope-labeled standards to MS^1^ samples. Moreover, quantitative MS sequencing is limited to oligomers up to DP 6 which need to be separated from residual polymeric substrates by filtration prior to derivatization.

In the following, we present a new setup of ultra high performance SEC coupled to RI and MS detection (UHPSEC-RI-MS) to characterize chitosan-cleaving enzymes, which combines the strengths of SEC coupled to RI for insights into the polymeric substrate, and of RI coupled to MS to analyze oligomeric products. On the one hand, we established an efficient separation of COS with protein UHPSEC columns, as reported before for oligomers of pectin^24^ or (oxidized) cellulose^25^, followed by detection using RI and MS (Sect. “Oligomer analytics”). Furthermore, challenges of COS quantification are addressed, making it possible to quantitatively analyze COS of different DP and FA without the need for filtration and derivatization. On the other hand, we demonstrate that UHPSEC-RI-MS can be used for MW analysis of chitosan polymers (Sect. “Polymer analytics”). By combining both approaches, oligomers and polymers can be analyzed in the same setup, providing the unique opportunity to simultaneously monitor the behavior of polymers during degradation and the resulting formation of oligomeric products. By taking consecutive live samples from a single ongoing reaction without additional sample preparation, this provides detailed insight into the course of an enzymatic reaction (results Sect. “Live digestion”): How fast is a polymer being degraded? How much of which COS are released? How does the product profile change over the course of the reaction?

While even live sampling of a single enzymatic reaction already gives helpful insights, analyzing a handful of samples with different enzymes or substrates allows a detailed comparison of the performance of various enzymes on the same substrate, or of one enzyme on different substrates. Although the latter would not require considerably more experimental effort, we decided to present only the data of a single enzymatic reaction using the very well-characterized chitosanase from *Bacillus* sp. MN (CsnMN)^18,26,27^. In this way, we want to emphasize how much information can be derived from a single run of our UHPSEC-RI-MS setup and how closely the results match the findings of previous, more laborious analyses of this enzyme.

## Materials and methods

### Polymers and oligomers

The chitosan polymers used to determine the relationship between *M*_w_ or *M*_n_ and retention time (RT) in the UHPSEC-RI-MS setup or as substrate for live digestion were kindly provided by Heppe Medical Chitosan (HMC; Halle, Germany) or Gillet Chitosan (Plumaudan, France). All chitosans were produced by heterogeneous deacetylation of crustacean chitins. The FA was determined by enzymatic MS fingerprinting^22^; *M*_w_, *M*_n_, and *Đ*_M_ were analyzed using SEC-MALS-RI^21^ (for details see Supplementary Table S1). Additionally, polymeric and oligomeric pullulan standards (Kit Pullulan, 10 × 0.1 g, Mp 342–739 000 Da, Polymer Standards Service, Mainz, Germany) as well as a mixture of chitosan oligomers derived from the acid hydrolysis of polymeric chitosan (661, kindly provided by Gillet Chitosan) were measured using UHPSEC-RI-MS. The latter contains basically all DPs ranging from the polymeric starting material (around 800) to the monomer, but distinct RI signals are only detected for DP 2–20. The *M*_w_ was given by the supplier for large pullulan standards or derived from the m/z values of MS spectra for chitosan or pullulan oligomers. To check their ionization efficiency and RI response, chitin oligomers of DP 2–6 (A2-6) were purchased from Megazyme (Wicklow, Ireland) and chitosan tetramers of different FA and PA were produced as described by Hembach et al.^28^.

### UHPSEC-RI-MS

Initially, the analytes were injected by an autosampler and separated using a Dionex Ultimate 3000RS UHPLC system (Thermo Fisher Scientific, Milford, USA) equipped with an ACQUITY UPLC Protein BEH SEC column (1.7 µm, 4.6 mm × 300 mm, Waters Corporation, Milford, USA). Either a column of 125 Å pore size (optimal separation range: 1–80 kDa) or one of 200 Å pore size (optimal separation range: 10–450 kDa) was used. Isocratic elution was performed at 40 °C with a flow rate of 0.4 mL/min^24^ and a runtime of 14 min using ammonium acetate (150 mM) and acetic acid (200 mM) dissolved in MilliQ water (final pH of 4.5) as solvent^21^. In general, polymer samples were dissolved in the solvent overnight, whereas oligomer samples were dissolved in MilliQ water, both at a final concentration of 1 g/L. For polymer and oligomer samples, 3 µL and 1 µL were injected, respectively. Following separation, the flow was split using a 1:1 splitter (Accurate, Dionex Corporation, Sunnyvale, USA). One half is used to measure RI signals with an ERC RefractoMax 520 (Thermo Fisher Scientific) at 10 Hz and 40 °C with recorder and integrator ranges of 512 µRIU and 125 µRIU/V, respectively. The other half flows into an electrospray ionization MS^n^ detector (amaZon speed; Bruker, Bremen, Germany) in positive mode with a capillary voltage of 4.5 kV, an end plate offset voltage of 500 V, a nebulizer pressure of 15 psi, a dry gas flow rate of 8 L/min, and a dry temperature of 180 °C. For spectra acquisition, the instrument was set to enhanced resolution scan mode with a target mass of m/z 700 covering a scan range of m/z 50–2000. The ICC target was set to 200,000 and the maximum accumulation time was 10 ms. Whereas the analysis of MS data was performed using Data Analysis 4.1 (Bruker) and an in-house Python script based on the module pymzML^29^, RI data were exported using Data Analysis 4.1 and evaluated using OriginPro 2023 (OriginLab, Northampton, USA). The latter is explained in more detail in the upcoming section.

### Live digestion

A single sample is composed of substrate, a suitable buffer, and an appropriate amount of enzyme. Pretests may be necessary to find a buffer type and concentration for appropriate enzyme activity, as well as to determine the amount of enzyme that will fit into the desired timeframe of the live digestion. Ideally, the first datapoints represent early timepoints of the reaction whereas the last timepoint represents (at least nearly) the endpoint. In the example shown in this work, chitosan 651 (Gillet Chitosan) with an FA of 0.22 and an *M*_w_ of about 130 kDa was dissolved at a concentration of 5 g/L in HCl (27.5 mM) and served as substrate (1 g/L in sample) in sodium acetate buffer (40 mM, pH 6 in sample). Recombinantly expressed chitosanase CsnMN^18,26,27,30^ was used for enzymatic hydrolytic cleavage (12.5 nM in sample).

**Figure 1:**
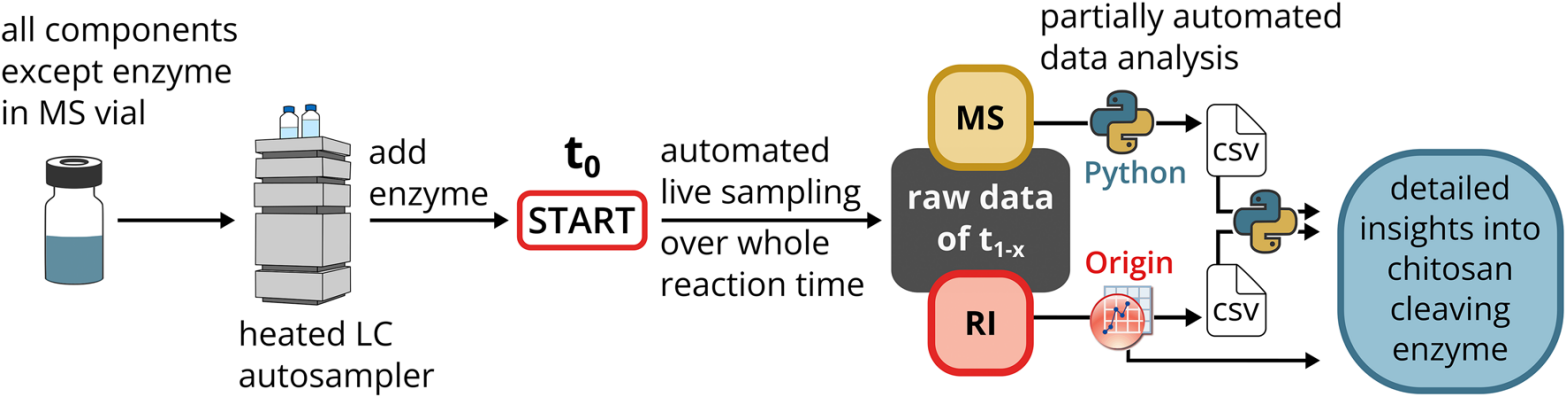
Workflow of a single live digestion experiment.

The workflow to perform a single live digestion experiment is summarized in Fig. 1. First, all sample components except the enzyme are prepared in an MS vial and moved to the autosampler, which is heated or cooled to the intended reaction temperature. As soon as the samples have reached this temperature, the reaction is started by adding the enzyme by manual pipetting or by using the autosampler. Immediately thereafter, the first sample representing t_0_ is taken from the reaction vial and injected into the UHPSEC-RI-MS setup with the 125 Å column as described above. If the time between enzyme addition and injection is too long, it is preferable to use a control without enzyme as t_0_. Now the autosampler can perform automated live sampling over the desired reaction time, resulting directly in raw MS and RI data for all chosen timepoints t_1-x_. In the example shown, we chose a total reaction time of 300 min at 37 °C and injected samples of 3 µL from the 50 µL reaction. Because the UHPSEC runtime was set to 14 min and the operations of the autosampler take one minute between runs, we sampled every 15 min. If certain timepoints are not desired, MilliQ water, other samples, or controls can be injected instead to avoid wasting sample. In case the solvent used for the UHPSEC differs in composition or pH from the reaction buffer inside the reaction vial, it is important to prevent contamination of the reaction vials with the solvent during injection. This can be achieved by changing the washing solution of the autosampler to water instead of connecting it to the solvent, and/or by using air bubbles within the autosampler needle to prevent the sample from mixing with the liquid in the sample loop.

Acquired data are analyzed partially automated: The MS data are converted to mzML format and processed with an in-house Python script based on the module pymzML^29^ to generate an output CSV file with the arbitrary intensity of the MS signal per oligomer and timepoint based on a list of chitosan oligomer candidates that can be found in the supplementary information (*candidate_file.csv*). In contrast, RI data are transferred to OriginPro 2023, plotted, and the areas of the peaks of each DP per timepoint as determined with the Peak Analyzer tool are copied to a second CSV file. Both CSV files are combined into a final output CSV file using another Python script (*ri_ms_combination.py*), which is available in the supplementary information, along with examples for the three CSV files and a user guideline for the Python script.

## Results and discussion

### Polymer analytics: UHPSEC-RI-MS as an alternative to SEC-MALS-RI

**Figure 2:**
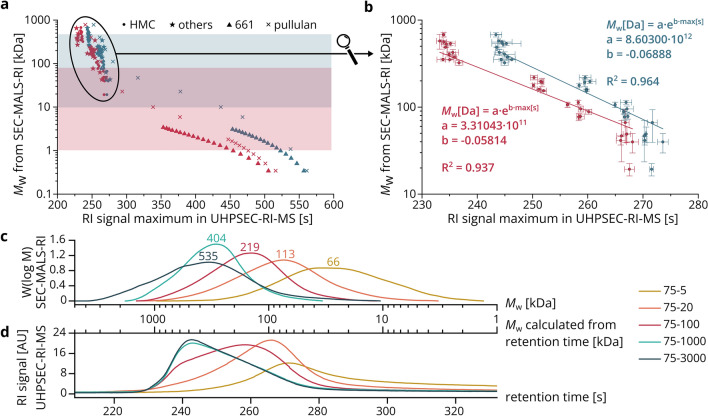
UHPSEC-RI-MS for the analysis of the *M*_w_ of chitosans. (**a**) Relationship between *M*_w_ determined by SEC-MALS-RI and RT of the RI signal maximum during UHPSEC-RI-MS. Chitosan polymers from HMC and other suppliers, and oligomers (661, Gillet Chitosan; included COS of DP 2–20 were considered) as well as pullulan standards ranging in *M*_w_ from 0.3–739 kDa were measured in the UHPSEC-RI-MS setup with two UHPSEC columns of different pore size, 125 Å (1–80 kDa optimum, red) or 200 Å (10–450 kDa optimum, blue). For smaller analytes, the MW was derived from the m/z values measured by MS; otherwise, the *M*_w_ was either given by the supplier for pullulan standards or determined by SEC-MALS-RI for chitosans. (**b**) Exponential relationship between *M*_w_ and RT of the RI signal maximum for chitosan polymers from HMC measured with a UHPSEC column of pore size 125 Å (red) or 200 Å (blue). (**c**) *M*_w_ distributions of selected HMC samples measured by SEC-MALS-RI, the average *M*_w_ calculated from this distribution (see Supplementary Table S1) is indicated above each curve. (**d**) *M*_w_ distributions of selected HMC samples calculated from the RI chromatograms of the UHPSEC-RI-MS setup (200 Å column) using the established relationship between *M*_w_ and RT.

The first step towards a simultaneous analysis of chitosan polymers and oligomers by UHPSEC-RI-MS was to establish our setup as an alternative to SEC-MALS-RI to determine the average MW of polymers based on newly established regressions. Figure 2a shows the relationship between the *M*_w_ determined by SEC-MALS-RI and the RT of chitosan polymers and oligomers as well as pullulan standards in the UHPSEC-RI-MS setup for two UHPSEC columns of different pore size. To facilitate an objective and easy data analysis, we defined the RT of the sample as the RT of the RI signal peak maximum. When comparing the performance of the two columns, separation is most efficient around 1 kDa or 80 kDa for the column with 125 Å or 200 Å pore size, respectively, which fits to the optimal MW ranges given by the supplier. As a consequence, resolving larger chitosan oligomers (DP 19 and 20 in the 661 sample) as well as the pullulans of *M*_w_ 0.5–2 kDa into individual peaks was only possible using the 125 Å column, resulting in more data points. Moreover, analytes generally elute faster from the 125 Å column, as expected from its smaller pore size. When comparing different analytes, the pullulan standards always elute later than corresponding chitosans of similar *M*_w_. This can be explained by the more globular structure of pullulans with their mixture of α-1,4 and α-1,6 linkages^31^ in contrast to the linear and rather stiff chitosans, making the former appear smaller during SEC. It is well known that the relationship between *M*_w_ and RT is different for different polysaccharides and that the calculation of *M*_w_ values, thus, must always be performed using regressions that are based on measurements of the same polysaccharide species. With the y-axis in logarithmic scale, exponential relationships between *M*_w_ and RT of chitosans appear as straight lines in the plot. It is not possible to find a suitable exponential equation to relate all *M*_w_ values to an RT, but for certain *M*_w_ ranges a common straight line is reasonable: the low *M*_w_ range from 0.1–1 kDa, the medium *M*_w_ range from 1–10 kDa, and the high *M*_w_ range from 10–1000 kDa. The set of polymeric chitosans (HMC samples) in the high *M*_w_ range from Fig. 2a is depicted as a close-up in Fig. 2b. Whereas samples of higher *M*_w_ exhibit low standard deviations and fit well to the regression, samples of low *M*_w_ deviate more. It should be mentioned that due to the low *M*_w_, the MALS signals of these samples are weak, which leads to inaccuracy and high deviations in the SEC-MALS-RI results. Based on the R^2^ values of the exponential regressions, the 200 Å column is more suitable for the analysis of chitosan polymers, although both columns give reliable results. An exponential relationship was also established between *M*_n_ and RT for both UHPSEC columns with R^2^ values above 0.96 (Supplementary Fig. S1).

The obtained exponential equations are used to convert signals from measurements of chitosan polymers in the UHPSEC-RI-MS setup to the corresponding *M*_w_. Figure 2c shows the *M*_w_ distribution obtained by SEC-MALS-RI of five selected samples; these were also measured by UHPSEC-RI-MS (200 Å column) and, based on the corresponding exponential equation, a second x-axis with the *M*_w_ was added to the chromatogram (Fig. 2d). On the one hand, the order of the samples’ elution times is the same for UHPSEC-RI-MS and SEC-MALS-RI, and in general, the *M*_w_ corresponding to the peak maximum in UHPSEC-RI-MS fits to the average *M*_w_ calculated from the SEC-MALS-RI distribution. On the other hand, limitations of the two methods become evident: At the high *M*_w_ end above 500 kDa, the UHPSEC-RI-MS setup is not suitable for efficient separation because samples 75/1000 (*M*_w_: 404 kDa) and 75/3000 (*M*_w_: 535 kDa) elute simultaneously even from the 200 Å column. Moreover, the *M*_w_ range above 500 kDa was not covered in the regressions. In contrast, SEC-MALS-RI shows different results for the two samples, as expected from viscosity data of the supplier, indicating a better performance of this method when analyzing very large polymers. However, for small polymers such as 75/5 (*M*_w_: 66 kDa), the MALS signal is barely higher than the baseline because the signal intensity decreases with decreasing MW, leading to high standard deviations. Hence, it is difficult to obtain reliable results on *M*_w_ and *M*_n_ by SEC-MALS-RI for low MW samples. On the contrary, UHPSEC-RI-MS separates small polymers well and is only dependent on the RI signal, making it the better solution at the lower *M*_w_ end. Not unexpectedly, it becomes apparent that considering only the single value for the average MW is a strong simplification when characterizing a chitosan sample—it is clearly important to always consider the *M*_w_ or *M*_n_ distributions from SEC-MALS-RI or UHPSEC-RI-MS as well.

As mentioned above, SEC-MALS-RI should still be the method of choice when analyzing very large polymers, at least when compared to the UHPSEC columns used in the UHPSEC-RI-MS setup here. Furthermore, it is obviously required to first establish the regression describing the relationship between RT in UHPSEC-RI-MS and MW of one type of analyte before MW analysis by UHPSEC-RI-MS is possible. However, once this is done, using UHPSEC-RI-MS has certain advantages over SEC-MALS-RI: Impurities or degradation products can directly be identified by MS. Moreover, data analysis is straightforward because the RI peak maximum is used to calculate the sample’s *M*_w_, whereas in SEC-MALS-RI the calculation is based on the integration of the RI and MALS peaks which can be challenging in case of low signal-to-noise ratios. Therefore, UHPSEC-RI-MS should be included in the general toolbox for chitosan polymer analytics as an alternative to SEC-MALS-RI. But more importantly here, the setup forms an integral part of the live digestion method described below (results Sect. “Live digestion”), addressing the polymer part of the sample.

### Oligomer analytics: prerequisites for semi-quantitative analysis

The second building block of this live digestion method is a simplified, quantitative oligomer analysis. The key to this is adequate separation of COS with the same UHPSEC columns used above for polymer MW determination. As discussed above, the results shown in Fig. 2a for COS (661) and pullulan oligomers indicate a better performance in oligomer separation for the UHPSEC column with a pore size of 125 Å compared to 200 Å, while maintaining reliable results in polymer analytics. Therefore, the 125 Å column was used in all further experiments. We were able to show that the efficiency of oligomer separation is not impaired by increased flow rates during chromatography (Supplementary Fig. S2). Hence, separation times can be reduced by increasing the flow rate as far as the pressure limit of the UHPSEC column allows without loss of oligomer separation performance. However, it should be noted that polymers of very high MW may be subject to shear degradation if the flow rate is too high, so the integrity of large polymers during UHPSEC should always be confirmed.

As mentioned in the introduction, the current main problem in the quantification of different COS is their different signal response factors in MS, which in turn derive from their different ionization efficiencies during electrospray ionization. To overcome this in the currently used quantitative MS sequencing method^23^, all D-units of COS are *N*-acetylated with isotope-labeled acetic anhydride prior to quantitative analysis. This derivatization converts non- or partially acetylated COS into chitin oligomers, while still being able to distinguish between original and reacetylated GlcNAc units by MS due to the isotope label. Combined with the addition of defined amounts of double isotope-labeled chitin standards of different DP, this allows absolute quantification of each COS of a certain DP and FA by MS. But the derivatization and the use of standards introduce limitations in the analysis of oligomers: Because double isotope-labeled chitin standards are only readily accessible up to DP 6, and fully acetylated COS are insoluble above DP 8, larger COS cannot be quantitatively analyzed using this method. Moreover, full *N*-acetylation requires prior removal of residual polymeric substrate and enzyme, making simultaneous analysis of polymers and oligomers within one sample or sampling live from an ongoing enzymatic reaction impossible.

In the following, we describe how the combination of MS and RI signals paves the way for a semi-quantitative analysis of chitosan oligomers without filtration and derivatization. This is only possible if three prerequisites are met: First, different oligomers of the same DP elute simultaneously; second, the RI signal intensity depends only on mass concentration; and third, the ionization efficiency of different oligomers of the same DP in MS is similar. The first prerequisite is ensured by a high ammonium acetate concentration in the SEC solvent, as shown in the supplementary information (Supplementary Fig. S3). In the following, the other two prerequisites are addressed.

**Figure 3:**
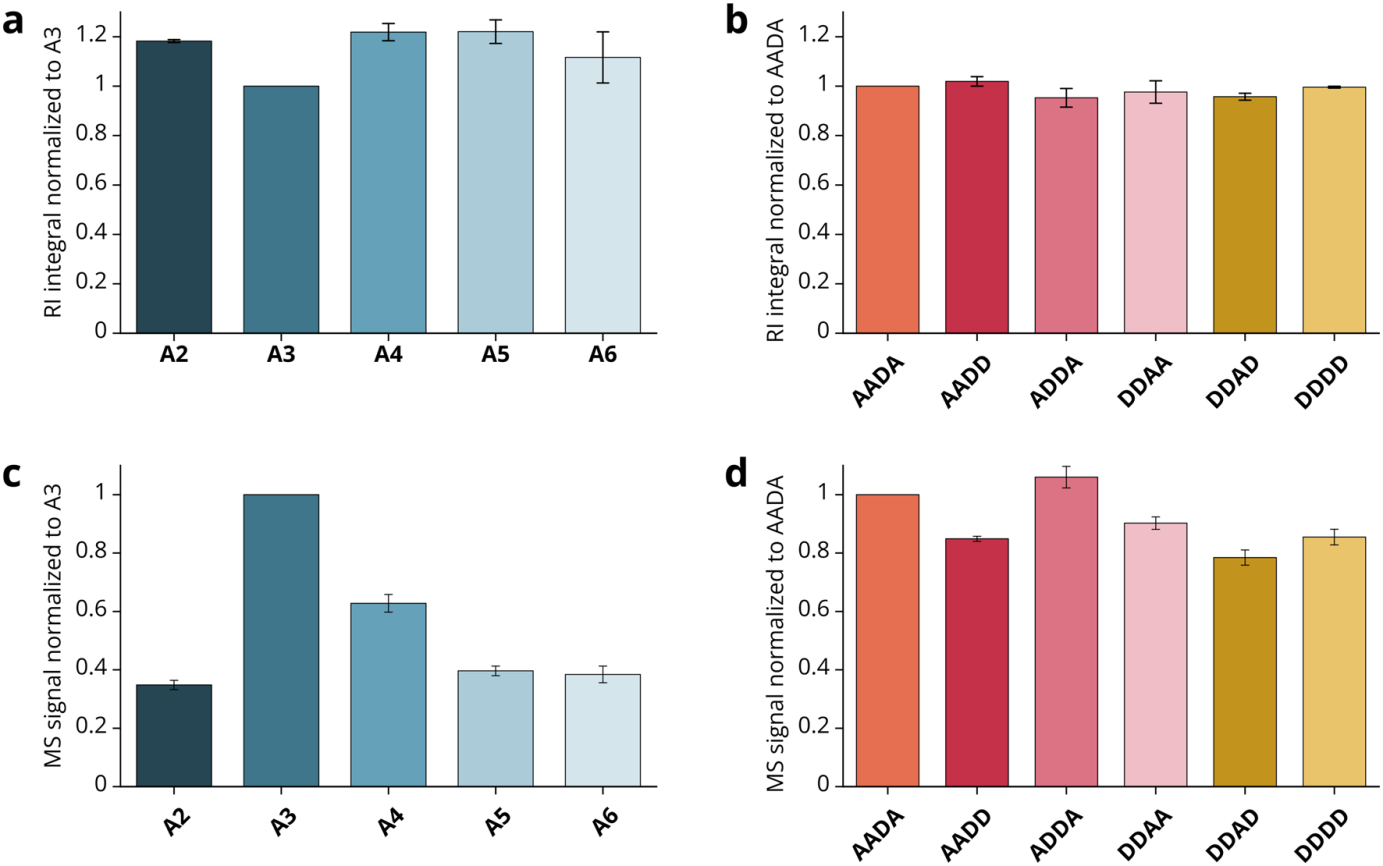
Response factors of different oligomers in RI and MS. All bars are averages of three analytical replicates with 1 µg of oligomer injected into the UHPSEC-RI-MS system. Ax describes chitin oligomers of DP x, if the PA of the tetramers is indicated, the monomer sequence is written out from non-reducing to reducing end. (**a**) Integrated RI peaks of COS of different DP normalized to A3. (**b**) Integrated RI peaks of tetramers of different FA and PA normalized to AADA. (**c**) Arbitrary intensities in MS of COS of different DP normalized to A3. (**d**) Arbitrary intensities in MS of tetramers of different FA and PA normalized to AADA. Exemplary base peak chromatograms and corresponding annotated MS spectra of all oligomers are shown in Supplementary Figure S4.

At a constant temperature, the RI shows a linear correlation with a compound’s mass concentration up to high concentrations^32^. This is commonly used in SEC-RI systems with constant temperature, solvent composition and flow rate for one analyte species to derive the relative mass concentration of the analyte from the RI peak integral. Indeed, under constant instrument parameters, the RI signal response is mainly dependent on the mass concentration, but the MW and response factor of the analyte (dn/dc value) also play a role^33^, resulting in an influence of the chitosans’ DP and FA. Even though this is neglected in most SEC-RI applications on chitosans anyway, we confirmed that neither the DP (Fig. 3a) nor the FA or PA (Fig. 3b) have a systematic or considerable influence on the RI signal response of oligomers. Hence, a dependency of the RI signal intensity on the chitosan oligomer mass concentration alone can be assumed.

In contrast, the signal response of chitosan oligomers in MS can differ dependent on the factors DP, FA or PA because of different ionization efficiencies during electrospray ionization. On the one hand, the ionization efficiency is vastly different for oligomers of different DP values (Fig. 3c): oligomers of DP 3 ionize best and are therefore overrepresented in the MS signal intensities compared to other DP values. On the other hand, oligomers of the same DP but with different FA and PA values possess similar ionization efficiencies, as shown in Fig. 3d.

The RI response factors allow the relative quantification of oligomers of one DP eluting in a single RI peak compared to oligomers of other DPs based on the integrals of the RI signal peaks. The MS response factors enable relative quantification of oligomers of one FA compared to oligomers of other FAs based on MS signal intensities, but only within one DP. A distinction between oligomers with the same DP and FA but different PA is not possible here. Combining both signals makes it possible to quantify each oligomer of a certain DP and FA relative to all others. In comparison to quantitative MS sequencing^23^, the combination of RI and MS proposed here does indeed result in a less accurate quantification, because it is based on the integration of RI peaks, because it does not provide absolute quantifications with internal standards, and most importantly, because the ionization of oligomers of the same DP with different FA is similar, but not identical—that is why we call it “semi-quantitative analysis”. However, our method is a unique way to easily quantify oligomers of defined DP and FA up to DPs with sufficient signal strength (typically at least up to DP 10) while simultaneously analyzing polymers within the same UHPSEC-RI-MS run. Combined with the fact that no further sample preparation is needed, this allows for easy and fast live analysis of chitosan-cleaving enzymes, as described in the following.

### Live digestion: fast and easy analysis of an enzyme’s action

The newly established methods for the analysis of polymers and oligomers by UHPSEC-RI-MS were finally combined into a live digestion method for the fast and easy characterization of chitosan-cleaving enzymes. A single injected sample measured with the UHPSEC-RI-MS setup already provides information on the amount, composition and average FA of oligomer products as well as on the amount and estimated *M*_w_ of the remaining polymer substrate. However, to get a complete picture of the enzyme activity on the substrate, it is necessary to monitor all these parameters over time. Because our method requires no sample preparation, samples containing substrate, enzyme and buffer can be injected directly. This makes it possible to sample automatically over time from a single vial using the autosampler. As shown in Fig. 1, the enzymatic digestion is started by adding the enzyme and from this timepoint on, the autosampler takes live samples from the enzymatic digestion and injects them directly into the UHPSEC-RI-MS system.

**Figure 4:**
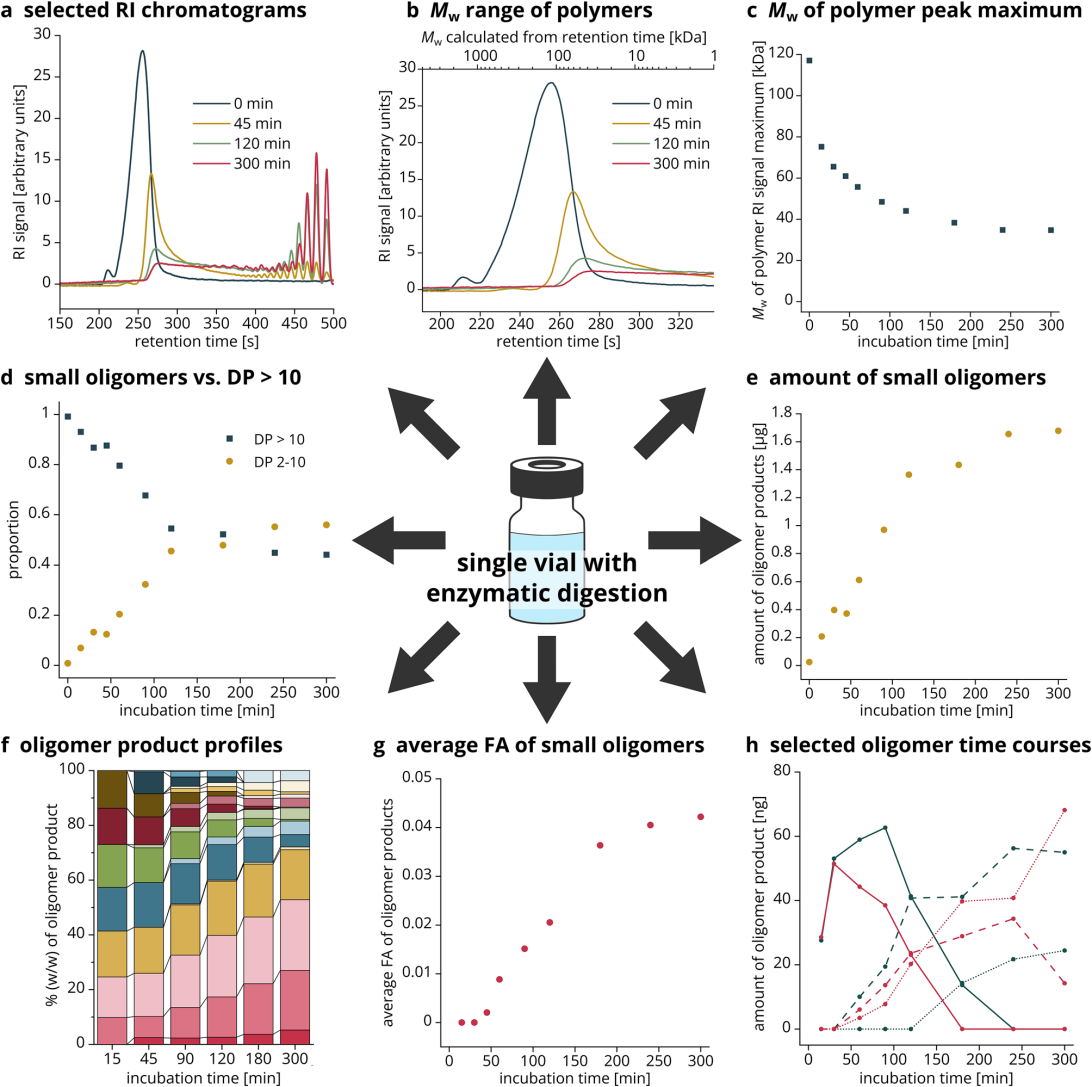
Data output of the UHPSEC-RI-MS setup from a single enzymatic live digestion. A chitosan polymer of FA 0.22 was incubated with chitosanase CsnMN for 300 min, separation was performed with the 125 Å column. (**a**) Selected raw RI chromatograms (more detailed in Fig. 5). (**b**) Polymer peak in RI chromatogram with *M*_w_ x-axis based on the relationship between *M*_w_ and RT established in Sect. “Polymer analytics”. (**c**) *M*_w_ of the polymer peak maximum in the RI chromatogram over time calculated based on the relationship between *M*_w_ and RT established in Sect. “Polymer analytics”. (**d**) Proportion of small oligomers (DP 2–10) vs. polymers and large oligomers (DP > 10) over time based on RI signal integrals. (**e**) Amount of small oligomers (DP 2–10) over time based on RI signal integrals and injected amount of chitosan (3 µg). (**f**) Profiles of weight percentages of small oligomer products (DP 2–10) over time (more detailed with legend in Fig. 6a). (**g**) Average FA of small oligomers (DP 2–10) over time based on weight percentages. (**h**) Selected time courses of oligomers as amount of product over time (more detailed with legend in Fig. 6b).

All data generated from a single vial with an enzymatic digestion are summarized in Fig. 4, representative of the digestion of a chitosan polymer (651, FA: 0.22, *M*_w_: 130 kDa) with the chitosanase CsnMN, sampled live over 300 min. We used DP 10 as cutoff between small oligomers on the one hand, and polymers and large oligomers on the other hand, because even at low concentrations of products, distinct RI peaks are formed for oligomers of DP ≤ 10.

**Figure 5:**
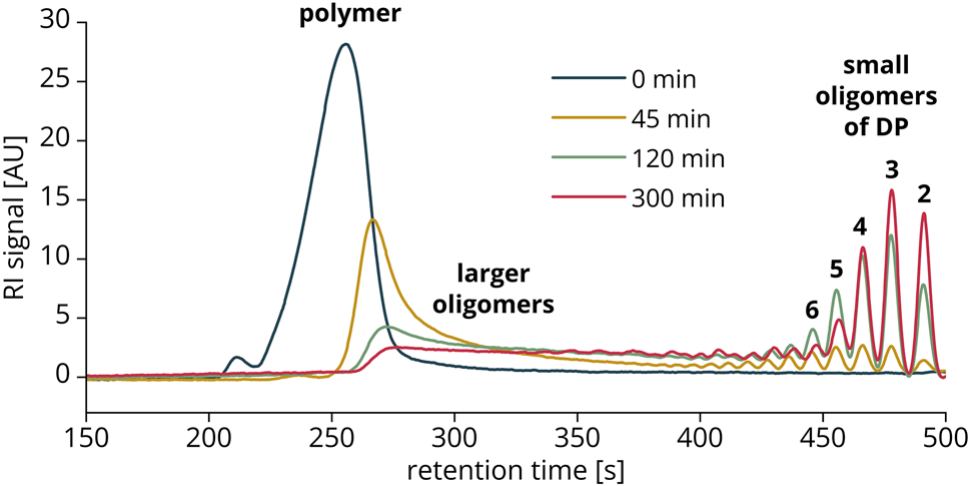
Selected RI chromatograms of the live digestion of FA 0.22 chitosan polymer with CsnMN. Shown are the raw RI data of four timepoints from the start until the end of the reaction.

First, the RI chromatograms (Figs. 4a, 5) should be considered, which allow to directly follow the cleavage of the polymers into oligomers. Whereas the substrate, as expected, contains only polymers, a considerable part was already hydrolyzed after 45 min to smaller polymers (shift of the peak towards later RT), large oligomers (tailing peak) and small oligomers (distinct peaks). Because an exo-enzyme would produce only small oligomers directly from the large polymer substrate, this indicates an endo-cleaving activity, which has already been reported for CsnMN^27^. After 120 min, the majority of the polymers was hydrolyzed and substantial amounts of small oligomers of DP 2–6 were produced. When comparing the chromatograms at timepoints 120 and 300 min, it becomes apparent that the enzyme continues to degrade oligomers of DP 5 and 6 to even smaller ones. The reaction was far progressed after 300 min, but considering the remaining polymers and large oligomers, probably not yet at its endpoint. To find the endpoint, one needs to continue the ongoing live digestion until the RI chromatograms show no more changes. Then it is useful to add fresh enzyme and take another sample after an additional incubation period to check if the reaction had stopped due to loss of enzyme activity or if the actual endpoint had been reached. At this point, only substrates remain that do not fit to the enzyme’s subsite preferences or required length, or the reaction has stopped due to product inhibition.

Based on the relationship between *M*_w_ and RT established by the regression in Sect. “Polymer analytics” (Fig. 2b), the *M*_w_ range of the polymers can be estimated as shown in Fig. 4b by introducing a second x-axis. To plot the cleavage of the polymers over the entire reaction time, the maximum of the polymer RI peak is converted to the corresponding *M*_w_ (Fig. 4c). Both figures show the hydrolytic cleavage of the polymers over time: Whereas the undigested substrate features a calculated *M*_w_ of about 120 kDa (SEC-MALS-RI of the substrate 651 had given an *M*_w_ of 134 kDa, Supplementary Table 1), the *M*_w_ of the RI maximum decreases over the course of the reaction—first rapidly, then more slowly, until it barely changes. At the same time, the RI peak integral of the polymer decreases drastically between the start of the reaction and 300 min, indicating efficient cleavage of the polymers.

The mass concentration-dependent integrals of the RI signals can further be used to quantify enzyme products of distinct size. Figure 4d shows the proportion of polymers and large oligomers (DP > 10) compared to small oligomers, with the latter fraction increasing and the former fraction decreasing linearly during the first 120 min. At this timepoint, half of the substrate has been cleaved into small oligomers of DP ≤ 10. Afterwards, the curves flatten, either because the enzyme lost activity over time or because the reaction was nearing its endpoint. Naturally, the same trend is visible when only the amount of the small oligomers in micrograms is plotted (Fig. 4e), which is obtained by multiplying the proportion of small oligomers per timepoint by the total amount of chitosan per injection, in this case 3 µg.

**Figure 6:**
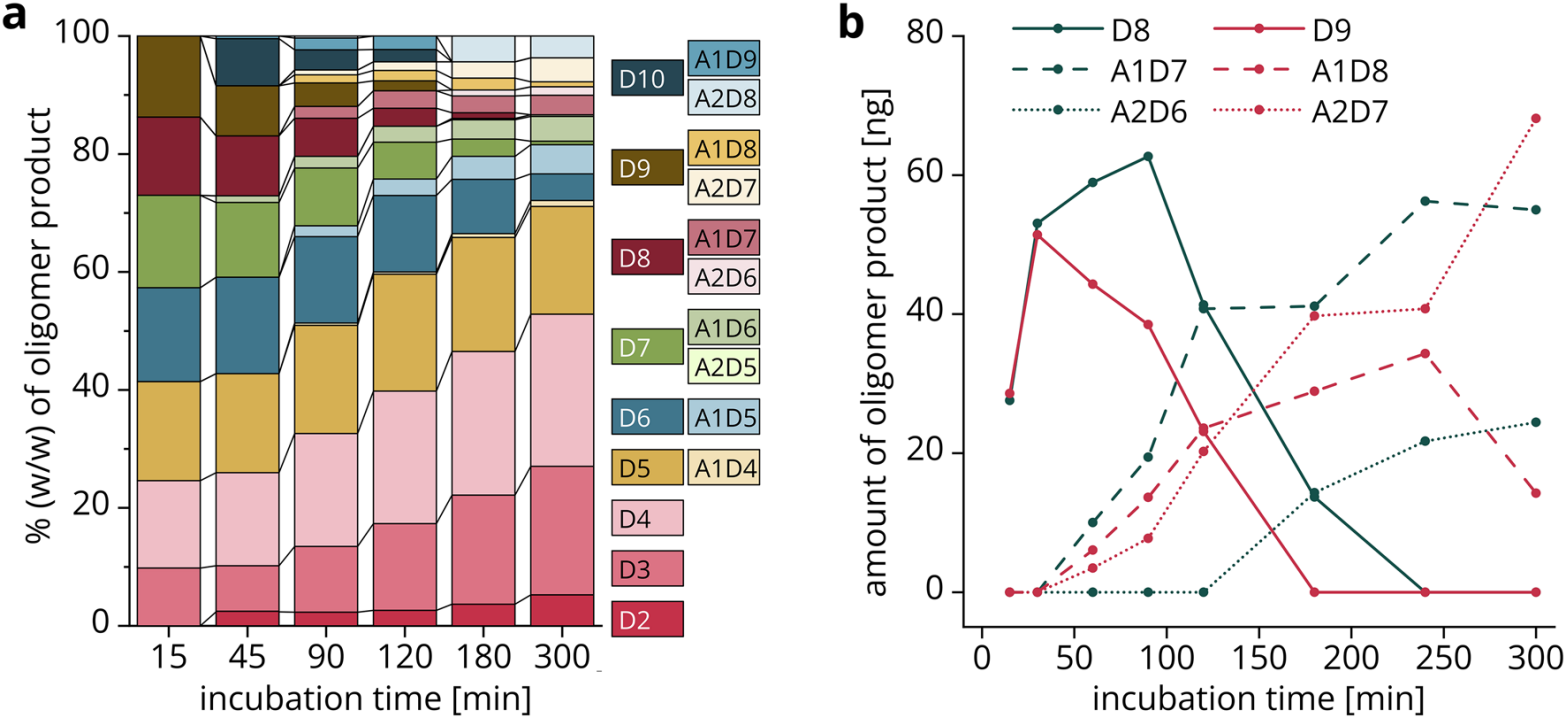
Amounts of oligomer products of specific DP and FA over time. Data are presented as (**a**) weight percentages of each oligomer product in stacked bars per selected timepoint, or (**b**) amount of selected oligomer products plotted over time. In both cases, the amounts are derived from the combination of RI signal integrals and MS signal intensities.

In a second data analysis step, the MS and RI data are combined. The obtained dimensionless amounts of each oligomer of a certain DP and FA can be plotted for each timepoint as oligomer product profiles (Figs. 4f, 6a), providing insight into the changing product composition over time. At the earliest timepoint of 15 min, all products are fully deacetylated (D3-D9). As the reaction progresses, two effects can be inferred from the product profiles: First, the large fully deacetylated oligomers D6-D10 were further cleaved into even smaller oligomers, resulting in a high share of DP 2–5 products at late timepoints. Second, the proportion of products above DP 5 with one or two acetyl groups increases, for example A1D5, A1D6 or A2D7. This is consistent with previous reports on CsnMN; the enzyme preferentially cleaves fully deacetylated sites of the substrate due to its high preference for D-units at subsites − 3 to + 3^27^, resulting in fully deacetylated products. After hydrolysis of these sites in the initial reaction phase, CsnMN increasingly binds to less acetylated sites because some of the enzyme’s subsites also accept A-units, but with lower affinity^18^. The resulting partially acetylated products are again no optimal substrates and accumulate. This increasing acceptance of acetylated units is also visible in Fig. 4g, where the average FA of the small oligomer products calculated from the product profiles is plotted over time. Based on the weight percentages of each oligomer shown in Figs. 4f and 6a and the absolute amounts of oligomer products shown in Fig. 4e, the total amount of individual oligomers can be followed over time (Figs. 4h, 6b). For example, D8 and D9 both increased initially, but were further cleaved into smaller oligomers as the reaction progresses. The longer the fully deacetylated substrate, the more likely the enzyme is to cleave; hence, the amount of D9 decreased earlier than that of D8. As for the product profiles and the average FA of oligomer products, the time courses of individual oligomers show an increasing acceptance of A-units. For DP 8, A1D7 started to accumulate after 60 min and even A2D6 was produced after 180 min. The longer the oligomer, the more likely is the presence of A-units in the product. Therefore, it is not surprising that both A1D8 and A2D7 were formed already after 60 min. A1D8 was degraded even further, as indicated by its decrease after 240 min.

Overall, our method simultaneously yields a host of information on a polymer cleaving enzyme, as exemplified and verified here using the example of the well-characterized chitosanase CsnMN: The enzyme is an endo-acting chitosanase with a strong preference for D-units, as described before^27^. Nevertheless, the enzyme tolerates A-units, especially once the strongly deacetylated parts of the substrate are already cleaved. This is in agreement with previous findings^18^ showing an almost absolute specificity for D-units at subsites − 2 to + 2 for the initial timepoints of the reaction, whereas A-units are accepted at subsites + 1 and + 2 only towards the endpoint of the reaction for more highly acetylated substrates. In contrast, the strong specificity for D-units at subsites − 1 and − 2 is maintained throughout different substrates and reaction times^18,26,27,30^. Because no partially acetylated oligomers of DP < 5 are produced, most A-units are accepted at subsites such as − 3 or + 3 rather than close to the catalytic site. Although still less than 20% of the sample was converted to smaller oligomers of DP ≤ 10 after 45 min, the *M*_w_ of the polymer peak was drastically reduced.

We want to emphasize that the selling point of our proposed method is the large amount of data providing a detailed overview of the enzyme’s actions that can be generated very quickly and with little experimental effort. Of course, the speed and convenience are traded off against accuracy, because the method is based on multiple approximations, each introducing a certain error: First, choosing the maximum of the RI signal peak for polymer MW analytics is easier but may be less accurate than the peak centroid, especially for asymmetric peaks. Second, the ionization efficiency of oligomers of the same DP but different FA and/or PA is similar, not identical, hence the term “semi-quantitative analysis”. And third, the exponential regressions for the relationship between *M*_w_ and RT of the RI signal maximum for chitosan polymers are not perfect, but approximations; moreover, the individual data points scatter, especially for low MW samples, due to low signals in SEC-MALS-RI measurements. Potentially, a more complex fitting model could give a better approximation and may even cover a wider MW range. Despite the imperfect accuracy, our proposed fast live digestion method is a valuable tool to provide detailed insight into an enzyme’s actions based on a single enzymatic digestion.

## Conclusion and outlook

In this study, we established UHPSEC-RI-MS as a suitable tool for MW analysis of chitosan polymers while simultaneously performing a semi-quantitative analysis of chitosan oligomers. Combined in the proposed live digestion method to characterize chitosan-cleaving enzymes, we can, i.a., follow the polymer cleavage over time, characterize and quantify the COS produced, and plot the changing product profile over the course of the reaction. Our goal was not to replace currently used methods for enzyme characterization—in particular, quantitative MS sequencing^23^ is still the only tool to analyze quantitative subsite preferences of enzymes. However, our UHPSEC-RI-MS setup offers a faster and easier analysis of many aspects than currently used methods. For example, many previously reported findings on CsnMN^18,26,27^ derived from various laborious experiments were obtained in this study from just a single live digestion experiment. This could enable mid-throughput screenings of different enzymes on the same substrate and/or the same enzyme on different substrates, each for multiple timepoints along the reaction. Apart from making analyses faster and easier, UHPSEC-RI-MS is the first method to analyze the degradation of the polymeric substrate along with the formation of oligomeric products, an aspect that has often been neglected.

In the setup presented here, the combined time for autosampler operations, separation and return to a stable baseline of one UHPSEC run is 15 min, so faster sampling is not possible. In case the enzyme in question has a very high turnover rate, one can either dilute the enzyme to achieve longer digestion times within this timeframe, or speed-up the UHPSEC run by using shorter UHPSEC columns and/or higher flow rates up to the maximum of the column’s pressure limit. The flow rate could be increased even further by increasing the column temperature. It should be noted, though, that speeding-up the UHPSEC may be accompanied by impaired separation performance and a potential shear degradation of large polymers at very high flow rates.

In addition to live measurements, the setup offers the possibility of live sampling and stopping the reaction for downstream processing. One would need to prepare vials with a suitable amount of acidic solution, and the autosampler could transfer sample into these vials in addition to injection into the UHPSEC-RI-MS system. The acidic conditions and volumes should be chosen such that the enzymatic reaction is stopped—the resulting samples could be *N*-acetylated and labeled for quantitative MS sequencing^23^ or used in bioactivity assays. Stopping under alkaline conditions is not suitable for chitosans, as chitosan polymers precipitate at high pH, unless this precipitation is intended in order to harvest only oligomeric products. In addition to chitosans, the UHPSEC-RI-MS setup may be suitable for the analysis of other polysaccharides. An interesting candidate is pectin, or more precisely homogalacturonan, a linear polysaccharide consisting of non-methylesterified and methylesterified galacturonic acid units. But for RI- and MS-based quantification to work, it must be confirmed that the ionization efficiency of pectin oligomers of the same DP is (almost) independent of their degree and pattern of methylesterification (Sect. “Oligomer analytics”). However, even if quantification by integration of RI and MS signals is not feasible for certain polysaccharides, the UHPSEC-RI-MS live digestion allows the analysis of any depolymerizing enzyme acting on soluble glycans over time—considering simultaneously the decomposing polymer and the formed oligomers.

### Supplementary Information

Supplementary Information.

## Data Availability

The full datasets generated and analyzed during this study are available from the corresponding author upon request.
