# Supplementary material for: Fast insights into chitosan-cleaving enzymes by simultaneous analysis of polymers and oligomers through size exclusion chromatography
Source: Sci Rep. 2024 Feb 10;14:3417. doi: 10.1038/s41598-024-54002-2 (PMC10858908; doi:10.1038/s41598-024-54002-2)
Supplement: Supplementary file 1 — Supplementary Information. [file 41598_2024_54002_MOESM1_ESM.zip › Supplementary information.pdf]

Supplementary information

Fast insights into chitosan-cleaving enzymes by simultaneous analysis of polymers and oligomers through size exclusion chromatography

Margareta J. Hellmann, Bruno M. Moerschbacher, Stefan Cord-Landwehr

Calculation and meaning of M_w, M_n and $Đ_M$

Unlike many samples of biological macromolecules such as proteins or nucleic acids, "true" polymer samples typically do not consist of molecules of a single molecular weight (MW), but are mixtures of molecules with different sizes. In case of chitosans, single molecules can differ in their degree of polymerization as well as their fraction (and pattern) of acetylation, resulting in a multitude of MWs. There are two standard measures to describe the average MW of polymer samples, the weight average MW (M_w) and the number average MW (M_n) which are calculated as indicated in equations (1) and (2), respectively. Whereas M_w is based on the weight fractions, M_n is based on the mole fraction of molecules in the sample. This means, the number of all molecules that are smaller than M_n is equal to the number of all molecules that are larger than M_n; similarly, the weight of all molecules that are lighter than M_w is equal to the weight of all molecules that are heavier than M_w. In other words, M_n weighs every molecule the same, regardless of its MW – this is the usual arithmetic mean, the way one would e.g. calculate the average height of a group of people. However, one could state that monomer units in smaller molecules are therefore overrepresented in the average value. In contrast, M_w weighs every molecule based on its MW when calculating the average value, thus, larger molecules contribute more strongly, inevitably resulting in M_w being higher than the corresponding M_n. Hence, the ratio of M_w by M_n is always > 1 and is termed the dispersity ($Đ_M$)[1] as given in equation (3). It quantifies the width of the MW distribution around the average; the smaller it is, the more uniform is the sample in terms of MW[2].

(1) $$M_w = \frac{\sum_i N_i {M_i}^2}{\sum_i N_i M_i}$$

(2) $$M_n = \frac{\sum_i N_i M_i}{\sum_i N_i}$$

(3) $$Đ_M = \frac{M_w}{M_n}$$

N_i: number of molecules of weight M_i in the sample
M_i: weight of a particular molecule of the sample

Similarly, dispersity can be used to describe the distribution of DPs in a polymer sample, with DP_w, DP_n and $Đ_{DP}$ (more generally termed X_w, X_n and $Đ_X$, respectively) defined *mutatis mutandis*[1]. It is noteworthy that owing to the different MW of the two monomeric units GlcN and GlcNAc, $Đ_M$ equals $Đ_{DP}$ only if the fraction of acetylation of the individual molecules is identical. Typically, chitosan samples with $Đ$ < 2 are considered to have a low dispersity. (Note that the polydispersity index (PDI) describing the distribution of particle sizes measured e.g. by dynamic light scattering is different from $Đ$. Whereas $Đ$ has no upper limit and $Đ$ = 1 indicates that all polymers have the same size, the PDI ranges between 0 and 1, with PDI = 0 signifying that all particles have the same size.)

Chitosan materials

Supplementary Table S1: Details on fraction of acetylation (FA) and MW of chitosan polymers used in this study. Listed are the FAs given by the supplier and those determined by enzymatic-mass spectrometric fingerprinting[3], as well as M_w, M_n and $Đ_M$ analyzed using SEC-MALS-RI[4]. Samples were kindly provided by (a) Heppe Medical Chitosan (HMC) or (b) Gillet Chitosan. HMC samples are named as X/Y where X represents the degree of deacetylation (= 1-FA [%]) and Y the viscosity [mPa·s] (typically higher viscosity is correlated with higher MW). *Averages of two biological replicates with two technical replicates each, the standard deviations are omitted here for clarity but included in corresponding plots.

sample	FA supplier	FA MS*	M_w*	M_n*	$Đ_M$*
70/3000[a]	0.30		559093	290660	1.93
70/1000[a]	0.30	0.26	351760	198688	1.94
70/100[a]	0.30	0.25	195975	94349	2.09
70/20[a]	0.30	0.23	95260	57391	1.74
70/5[a]	0.30		39777	21654	2.46
75/3000[a]	0.25		534895	257705	2.09
75/1000[a]	0.25		404073	229560	1.79
75/100[a]	0.25		218508	127000	1.72
75/20[a]	0.25	0.24	113078	58496	2.02
75/5[a]	0.25		66342	20233	3.17
80/3000[a]	0.20		678755	283975	2.41
80/1000[a]	0.20		373883	211688	1.77
80/100[a]	0.20		192655	100549	1.92
80/20[a]	0.20	0.17	106340	79159	1.36
80/5[a]	0.20	0.11	19319	8462	2.85
85/3000[a]	0.15		573875	209360	2.73
85/1000[a]	0.15		350473	218023	1.63
85/100[a]	0.15		177278	99350	1.81
85/20[a]	0.15	0.09	78019	58336	1.39
85/5[a]	0.15		41370	14638	2.93
90/3000[a]	0.10		527685	255723	2.07
90/1000[a]	0.10		424413	264315	1.62
90/100[a]	0.10		159808	113990	1.41
90/20[a]	0.10	0.08	77028	53493	1.44
90/5[a]	0.10		48958	15542	3.43
95/3000[a]	0.05		486490	273945	1.78
95/1000[a]	0.05		322548	205415	1.58
95/100[a]	0.05		154238	90844	1.72
95/20[a]	0.05	0.03	88476	32130	2.83
95/5[a]	0.05		46739	11109	4.20
651[b]	0.20	0.22	133860	85261	1.57

Relationship between M_n and retention time

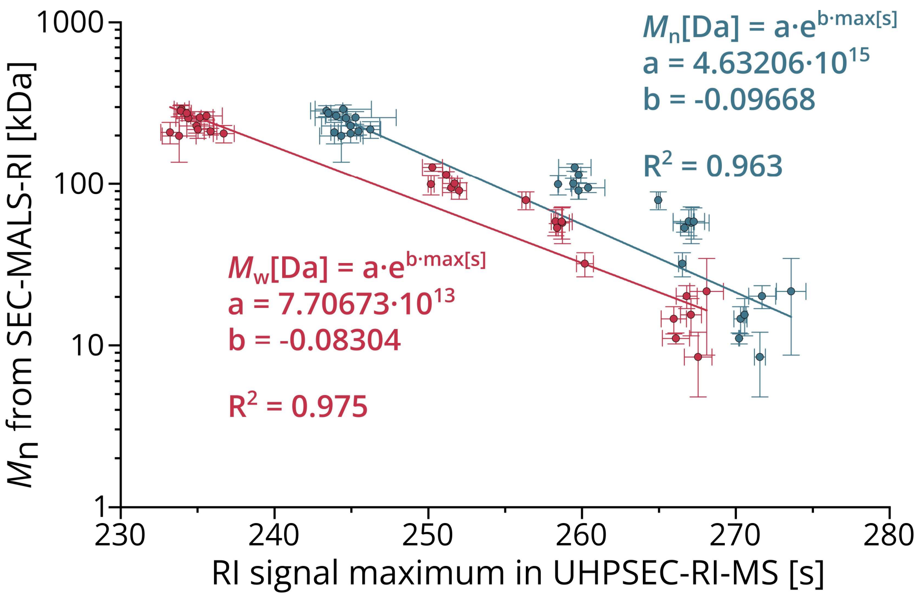

Supplementary Figure S1: UHPSEC-RI-MS for the analysis of the M_n of chitosans. Exponential relationship between M_n and the retention time of the RI signal maximum for chitosan polymers from HMC measured with a UHPSEC column of pore size 125 Å (red) or 200 Å (blue).

UHPSEC performance at different flow rates

Analyzing the oligomer region of UHPSEC-RI-MS chromatograms allows a straightforward evaluation of the influence of different parameters on separation performance. Our findings indicate that the retention time of a compound is solely dependent on the volume of solvent flown (see Supplementary Fig. S2), thus, doubling the flow rate results in exactly half the retention time. High flow rates do not impair the separation performance, therefore the pressure limit of the UHPSEC column is the only factor limiting the flow rate and hence, the reduction in separation times.

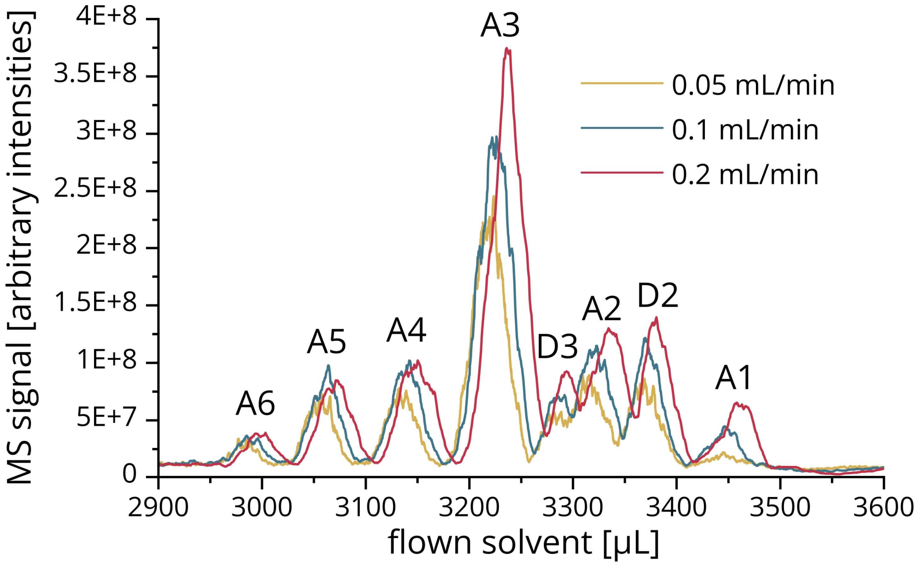

Supplementary Figure S2: Separation performance of the UHPSEC-RI-MS setup at different flow rates. MS signals of GlcNAc standards of DP 1-6 (A1-6) or GlcN standards of DP 2-3 (D2-3) separated with a UHPSEC column of 125 Å pore size using three different flow rates (0.05, 0.1 or 0.2 mL/min).

Influence of ammonium acetate concentration in SEC solvent

When separating chitosan oligomers based on size, the size difference of 42 Da between heavier A- and lighter D-units should be taken into consideration, especially for very small oligomers such as dimers and trimers. For SEC solvents with low levels of ammonium acetate (50 mM), non-acetylated oligomers like D2 or D3 elute significantly later than acetylated ones with the same DP (see Supplementary Fig. S3). In comparison, increased concentrations of ammonium acetate (200 mM) lead to a shift of (partially) deacetylated oligomers to earlier retention times whereas fully acetylated COS elute as before. Importantly, only at the higher acetate concentration, the smallest trimer (D3) elutes before the largest dimer (A2) and the smallest dimer (D2) elutes before the largest monomer (A1). In contrast, D3 elutes after A2 while D2 and A1 co-elute for the lower acetate concentration. Possibly, the negatively charged acetate ions interact with the positively charged protonated amino functions of D-units, which increases their hydrodynamic volume making them more similar in size to A-units. A high acetate concentration in the SEC solvent is therefore useful to ensure a more purely DP-dependent, nearly simultaneous elution of oligomers of one DP but different FAs, resulting in one RI peak per DP – a prerequisite for the quantification in our proposed method. It works especially well for products of chitosan-cleaving enzymes which normally exhibit limited variation in their FAs due to the enzymes' preferences for A- or D-units.

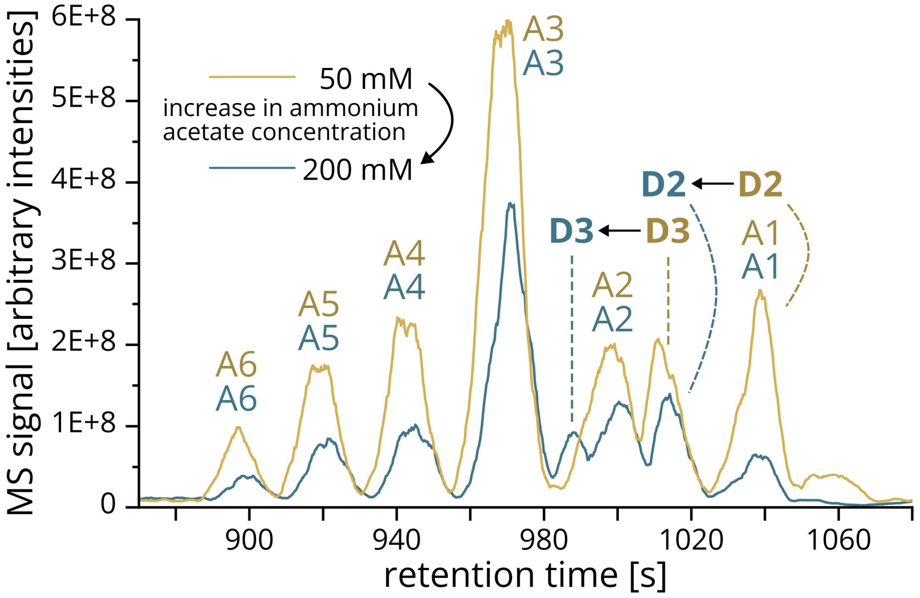

Supplementary Figure S3: Influence of the ammonium acetate concentration in the solvent on oligomer retention times. MS signals of GlcNAc or GlcN standards (A1-6 and D2-3, respectively) separated with a UHPSEC column of 125 Å pore size using two different ammonium acetate concentrations in the solvent, 50 mM (yellow) or 200 mM (blue).

Exemplary MS spectra

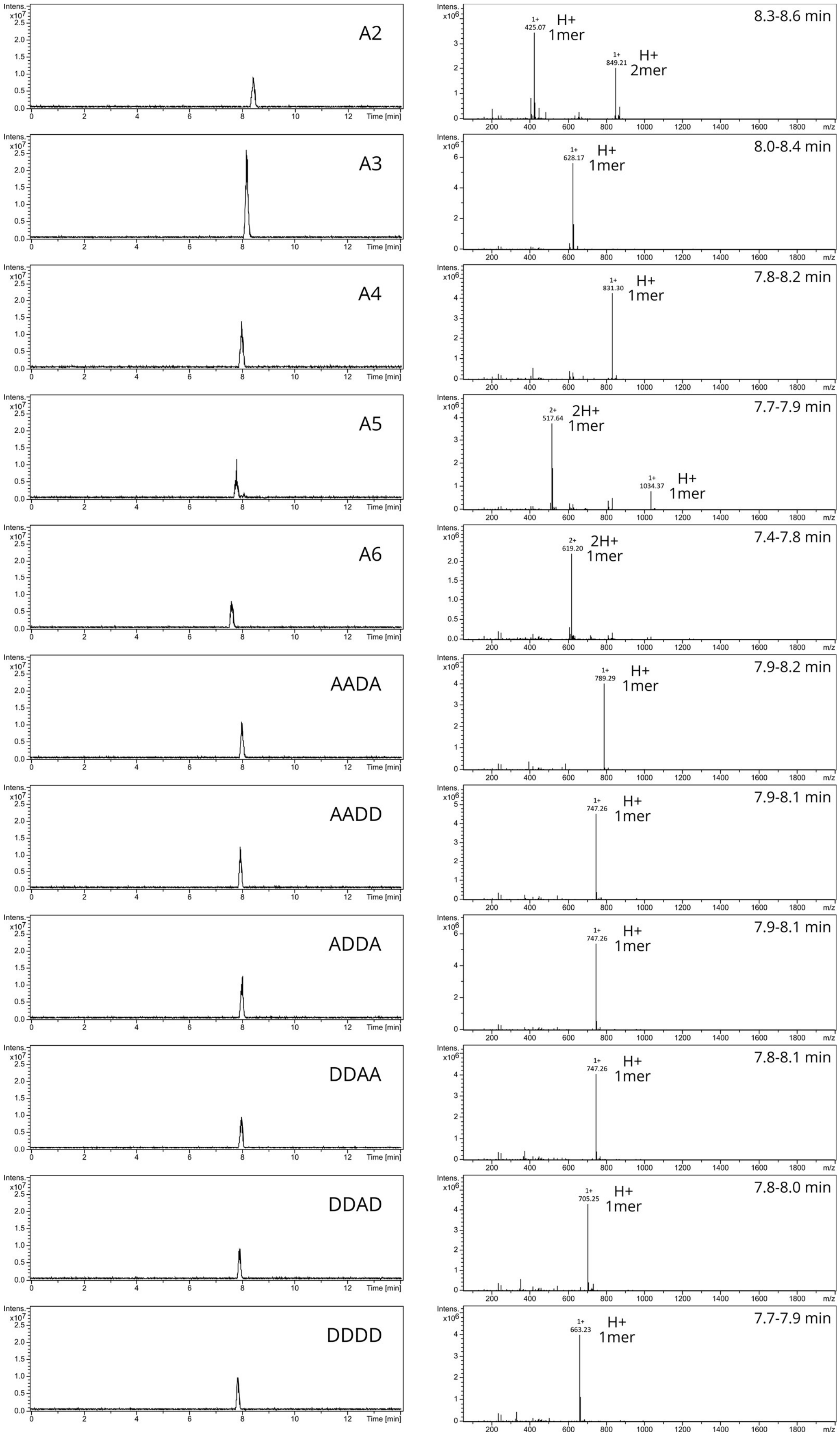

Supplementary Figure S4: Exemplary base peak chromatograms (left) and MS spectra (right) of oligomers measured by UHPSEC-RI-MS. The injected oligomer is indicated in the chromatogram, the corresponding MS spectrum of the peak averaged over the indicated timespan is shown with annotation of the m/z peaks belonging to the oligomer.

Automated analysis of RI and MS data

To process the large MS datasets, we use an in-house Python script based on the module pymzML[5] to quantify arbitrary intensities of the different oligomers per sample. The script identifies eluting compounds through peak picking and annotates chitosan oligomers based on their m/z values and retention time. This is performed based on candidate files containing all selected chitosan oligomer candidates in different adducts and charge states that may occur in the sample. The candidate file used throughout this study can be found in the Supplementary information (*candidate_file.csv*) and contains all possible oligomers of DP 2-10 and FA 0-1 as proton adducts (charge states +1, +2 and/or +3 depending on the DP).

The Python script *ri_ms_combination.py* available in the Supplementary information can be used to automatically combine RI and MS data, both in CSV format. An RI CSV file contains different samples in rows, and the integral of the RI signal per DP obtained from Origin 2023 in columns (see *RI_data.csv*). An MS CSV file (see *MS_data.csv*) also lists the different samples in rows which need to have the same names as in the RI CSV file; the columns contain the MS signal in arbitrary signal intensities of the different oligomers. The script needs to be executed with Python 3. If the RI and MS CSV files are in the current directory and have the default names *MS_data.csv* and *RI_data.csv*, respectively, executing the script requires no additional arguments:

```
>python3 ri_ms_combination.py
```

If the CSV files are in a different directory and/or have different names, file paths can be specified using the options --ri and/or --ms as follows:

```
>python3 ri_ms_combination.py --ri “path to RI file\RI_file.csv” --ms “path to MS file\MS_file.csv”
```

The script *ri_ms_combination.py* will create an output CSV file named *RI_MS_data_combined.csv* in the current directory, unless the user specifies a different name using the --output option. Again, the rows list the different samples, the columns contain the dimensionless relative amounts of each oligomer which can easily be converted into weight percentages of each oligomer per sample.

References

1. Gilbert, R. G. *et al.* Dispersity in polymer science (IUPAC Recommendations 2009). *Pure Appl. Chem.* **81**, 351–353; doi:10.1351/PAC-REC-08-05-02 (2009).
2. Shrivastava, A. Polymerization. in *Introduction to Plastics Engineering* 17–48; doi:10.1016/B978-0-323-39500-7.00002-2 (Elsevier, 2018).
3. Wattjes, J., Niehues, A. & Moerschbacher, B. M. Robust enzymatic-mass spectrometric fingerprinting analysis of the fraction of acetylation of chitosans. *Carbohydr. Polym.* **231**, 115684; doi:10.1016/j.carbpol.2019.115684 (2020).
4. Schatz, C., Viton, C., Delair, T., Pichot, C. & Domard, A. Typical Physicochemical Behaviors of Chitosan in Aqueous Solution. *Biomacromolecules* **4**, 641–648; doi:10.1021/bm025724c (2003).
5. Kösters, M. *et al.* pymzML v2.0: introducing a highly compressed and seekable gzip format. *Bioinformatics* **34**, 2513–2514; doi:10.1093/bioinformatics/bty046 (2018).
